# Ego-resiliency moderates the risk of depression and social anxiety symptoms on suicidal ideation in medical students

**DOI:** 10.1186/s12991-022-00399-x

**Published:** 2022-06-18

**Authors:** Eun Hyun Seo, Hae-Jung Yang, Seung-Gon Kim, Hyung-Jun Yoon

**Affiliations:** 1 Premedical Science, College of Medicine, Chosun University, Gwangju, Republic of Korea; 2 Department of Psychiatry, Chosun University Hospital, Chosun University, Gwangju, Republic of Korea; 3 Department of Psychiatry, College of Medicine, Chosun University, 309 Pilmun-daero, Gwangju, Dong-gu 61452 Republic of Korea

**Keywords:** Suicidal ideation, Ego-resiliency, Self-esteem, Social support, Medical students

## Abstract

**Background:**

Little is known about the role of protective factors in suicidal ideation among medical students. This study aimed to examine the association between suicidal ideation and protective (self-esteem/ego-resiliency/social support) and risk (depression/social anxiety) factors.

**Methods:**

Data on sociodemographic factors, depression, social anxiety, self-esteem, ego-resiliency, social support, and current suicidal ideation were collected from 408 medical students. A logistic regression model was constructed to identify the independent impact of potential influencing factors on suicidal ideation. Potential moderating effects were also explored.

**Results:**

Thirty-eight participants (9.3%) reported experiencing suicidal ideation. Younger age, higher levels of depression, social anxiety, and lower levels of self-esteem, ego-resiliency, and social support were found to be significantly correlated with suicidal ideation. In the final model, higher levels of depression and social anxiety were associated with an increased risk of suicidal ideation, while higher levels of self-esteem and social support were associated with a decreased risk of suicidal ideation. Although the independent effect was not significant, the interactions of ego-resiliency with both depression and social anxiety on suicidal ideation were significant. Higher levels of ego-resiliency acted as a buffer against suicidal ideation among those with higher levels of depression or social anxiety.

**Conclusions:**

In addition to risk factors, this study revealed the underlying protective and moderating factors of suicidal ideation among medical students. Mental health programs focusing on enhancing ego-resiliency, self-esteem, and social support may contribute to suicide prevention in medical students.

## Introduction

Despite many efforts toward reducing suicide, the suicide rate has increased by more than 50% worldwide in the past half-century [1]. In particular, the suicide rate in Korea is the highest in the Organisation for Economic Cooperation and Development (OECD) countries, with the death rate due to suicide per 100,000 persons being 24.6 in 2019 [2]. A history of suicide attempts is known to be a strong predictor of later attempts and suicide completion [3]. Also, suicidal ideation and planning have been regarded as important steps in the process of suicide, characterized by a stepwise hierarchy of behaviors with an underlying gradient of severity [4]. A previous study found that the severity of past suicidal ideation was the most important predictor differentiating suicide attempters from suicide non-attempters in patients with depression, even after controlling for other variables, suggesting that severe suicidal ideation should not be ignored [5]. Moreover, suicidal ideation has been reported as a marker of extreme psychological distress, such as desperation [6].

College entrance can be stressful for students who are psychologically vulnerable and have poor social support because this period requires various duties and major decisions for their future. Medical students are at high risk for suicidality, and psychological distress, such as depression and burnout, has been associated with suicidal ideation [7, 8]. In a previous review [9], levels of overall psychological distress were significantly higher in medical students than in the general population and age-matched peers. A variety of factors during medical education, including academic pressure, sleep deprivation, workload, and exposure to patients’ death and suffering, have been hypothesized to contribute to this distress [9]. Meanwhile, medical students are less likely to receive appropriate treatment than the general population, despite apparently better access to care [10]. Stigmatization or fear of confidentiality related to the use of mental health services could act as a barrier to seeking help, which may potentially increase suicide risk in this population.

Among various psychiatric symptoms, depression and social anxiety among medical students have been underlined because these symptoms are common and associated with impaired academic performance as well as poor quality of life [11, 12]. Depression is the most frequent psychiatric symptom associated with suicidal ideation, whereas an association between suicidal ideation and social anxiety has not been reported. In a previous study among undergraduate students [13], depression significantly affected suicidal ideation through burdensomeness. In contrast, social anxiety was more distally associated with suicide risk through thwarted belongingness. In addition to these clinical symptoms, sociodemographic and other factors such as financial difficulty, a history of drug use, feeling neglected by parents, and dissatisfaction with academic performance have been associated with suicidal ideation among medical students [14]. However, focusing on individual risk factors may be insufficient to prevent suicide because of the relatively small portion of the variance and lack of specificity [15]. In fact, some risk factors are difficult to adjust.

Recently, protective factors have received increased attention as an alternative approach in research on suicidal behavior given the limitations of individual risk factors. In general, protective factors can be divided into internal and external components. Self-esteem and ego-resiliency are included in the internal component, whereas social support is an external component. Self-esteem refers to the extent to which a person values, approves, or likes oneself. Ego-resiliency is defined as a flexible and resourceful adaptation or capacity to adapt one’s behavior to ever-changing situations and environmental demands [16]. High levels of self-esteem and ego-resiliency have shown a significant protective effect on depression in adolescents [17, 18], implying that these factors may play an important role in the prevention of suicidality. In addition, social support has been negatively associated with suicidal ideation among veterans [19]. Thus, it is possible that self-esteem, ego-resiliency, and social support may protect against suicidal ideation among medical students.

Most studies on suicidal ideation among medical students have explored the prevalence and risk factors of suicidal ideation. Meanwhile, despite evidence regarding the meaningful effects on psychiatric symptoms, there have been only a few studies investigating the impact of protective factors on suicidal ideation among medical students. Further, the moderating and independent effects of protective factors on suicidality need to be investigated, because a certain protective factor may have a significant indirect effect on suicidal ideation through the interaction with risk factors. Moreover, there are limited studies on the prevalence and factors related to suicidal ideation in this population in Korea. For these reasons, we examined the impact of protective factors (self-esteem, ego-resiliency, and social support) and risk factors (depression and social anxiety) on suicidal ideation in a sample of medical students.

## Methods

This study was conducted with 408 medical students at Chosun University in Gwangju City, Korea, from September 2019 to December 2019. The students were participants in another study that has been published recently and in which the procedures for data collection are described [20]. Briefly, participants completed a self-report questionnaire on sociodemographic factors (age, gender, year in school, marital status, living situation, religion, subjective socioeconomic status, and subjective amount of pocket money), protective factors (self-esteem, ego-resiliency, and social support), risk factors (depression and social anxiety), and suicidal ideation. This study was approved by the Institutional Review Board of Chosun University.

### Measures

#### Depressive symptoms

Depressive symptoms were assessed by the Beck Depression Inventory (BDI) [21]. The BDI is a 21-item instrument designed to evaluate the severity of depressive symptoms. Each item is rated from 0 to 3, and the total score ranges from 0 to 63, with a higher score representing more severe depressive symptoms. In the present study, the total score of the BDI that omitted item 9 for suicidal ideation was used to avoid circularity in both univariate and multivariate analyses. The validity and reliability of the BDI in the Korean population have been confirmed previously [22]. Cronbach’s alpha for the BDI was 0.90 in this sample.

#### Suicidal ideation

Presence of current suicidal ideation was assessed by the response to item 9 on the BDI. Responses were divided into two groups: non-suicidal ideation (0) and suicidal ideation (1–3).

#### Social anxiety symptoms

Social anxiety symptoms were evaluated using the Social Phobia Inventory (SPIN), which is a screening instrument for identifying social anxiety [23]. It is composed of 17 items in three symptom domains of social anxiety (i.e., fear, physical symptoms, and avoidance). Each item is rated on a 5-point scale (from 0 to 4). The total score ranges from 0 to 68, with a higher score reflecting greater social anxiety symptoms. The reliability and validity of the SPIN have been confirmed in Korea [24]. Cronbach’s alpha for the SPIN was 0.93 in the present study.

#### Self-esteem

Self-esteem was assessed using the Rosenberg Self-Esteem Scale (RSES) [25]. The RSES is a 10-item self-report questionnaire with five positive and five negative items reflecting high and low self-esteem, respectively. Each item is rated from 1 (strongly disagree) to 4 (strongly agree), but negative items are reverse-scored. The total score ranges from 10 to 40, with a higher score indicating a higher level of self-esteem. The reliability and validity of the Korean version of the RSES have been confirmed [26]. In this study, Cronbach’s alpha was found to be 0.88.

#### Ego-resiliency

Ego-resiliency was measured using the Ego-Resiliency Scale (ERS) developed by Block and Kremen [16]. The Korean version of the ERS translated by Yoo and Shim was used in this study [27]. The scale is composed of 14 items that assess flexibility, curiosity, generosity, and social skills. Items are rated on a 4-point scale ranging from 1 (does not apply at all) to 4 (applies very strongly). The total score ranges from 14 to 56, with a higher score reflecting a higher level of ego-resiliency. In the study by Yoo and Shim, Cronbach’s alpha was 0.67. In this study, Cronbach’s alpha was found to be 0.83.

#### Social support

The Duke-University of North Carolina Functional Social Support Questionnaire (Duke-UNC FSSQ) was used to assess the level of social support [28]. Eight items from the Duke-UNC FSSQ, consisting of two subscales (Confidant Support and Affective Support), were used to yield the mean score for social support [29]. The Confidant Support subscale (5 items) evaluates the degree of confidant support, such as opportunities to talk to someone about personal problems. The Affective Support subscale (3 items) evaluates the degree of emotional support and care from family and friends. Each item is rated from 1 (much less than I would like) to 5 (as much as I would like), with a higher score indicating a higher level of perceived social support. The Korean version of the Duke-UNC FSSQ has shown high reliability and moderate validity [30]. In this study, Cronbach’s alpha was found to be 0.93.

#### Statistical analysis

Participants were grouped into either a suicidal ideation group or a non-suicidal ideation group based on their BDI item 9 score. Continuous variables were checked for normal distribution using the Kolmogorov–Smirnov test. Since all continuous data were non-normally distributed, nonparametric tests were applied for the analysis. Sociodemographic, protective (self-esteem, ego-resiliency, and social support), and risk (depression and social anxiety) factors were examined for their association with suicidal ideation using the Mann–Whitney *U* test for continuous variables and the Chi-square test or Fisher’s exact test for categorical variables. Sociodemographic, risk, and protective factors that were significantly different between the groups in the univariate analyses were selected as independent variables in the logistic regression model. Before entering the model, protective and risk factors were transformed into dichotomous variables (high and low) using receiver operating characteristic (ROC) curves to select the cut-off point with the best discrimination capability. A test of the null hypothesis that the area under the curve (AUC) was 50% was performed using the Wilcoxon rank sum test, and cut-off points were calculated based on the Youden index (*J*) [31]. The point with the largest *J* value was defined as the optimal point. A multiple logistic regression model using the backward-conditional method was used to identify factors associated with suicidal ideation. To explore the potential moderating effects, a Chi-square automatic interaction detection (CHAID) [32] with a Bonferroni correction was performed in cases where it was necessary. Significance was set at *p* < 0.05 (two-tailed) for all tests. All statistical analyses were performed using SPSS version 26.0 for Windows (SPSS Inc., Chicago, IL, USA).

## Results

A total of 418 medical students participated in the study. Excluding 10 invalid questionnaires (those with > 25% questions unanswered), the data from 408 students were included in the analyses. Of the 408 participants, 255 (62.5%) were men and 153 (37.5%) were women. Age ranged from 20 to 45 years, and the mean age was 26.3 ± 4.4 years. Overall, 38 (9.3%) participants reported experiencing suicidal ideation. Participants with suicidal ideation were significantly younger than those without suicidal ideation. However, there were no significant differences with regard to the other sociodemographic characteristics. Although statistically non-significant, the proportion of participants with suicidal ideation tended to be higher in first- and second-year students than in the third- and fourth-year students. The prevalence of suicidal ideation was highest among first-year students (*n* = 16, 13.3%), followed by second-year (*n* = 12, 12.8%), fourth-year (*n* = 5, 5.3%), then third-year students (*n* = 5, 5%). The sociodemographic characteristics of the sample and the comparisons according to the presence of suicidal ideation are shown in Table 1.

**Table 1:** Group comparisons of sociodemographic characteristics according to the presence of suicidal ideation

Sociodemographic characteristic	Suicidal ideation		Total *N* (%) or mean ± SD 408 (100.0)	*χ*^2^	*p*
	No *N* (%) or mean ± SD 370 (90.7)	Yes *N* (%) or mean ± SD 38 (9.3)			
Age	26.4 ± 4.4	25.0 ± 4.2	26.3 ± 4.4		0.043^†^
Gender					
Male	236 (63.8)	19 (50.0)	255 (62.5)	0.01	0.904
Female	134 (36.2)	19 (50.0)	153 (37.5)		
Year					
First year	104 (28.1)	16 (42.1)	120 (29.4)	7.60	0.055
Second year	82 (22.2)	12 (31.6)	94 (23.0)		
Third year	95 (25.7)	5 (13.2)	100 (24.5)		
Fourth year	89 (24.1)	5 (13.2)	94 (23.0)		
Marital status					
Never married	350 (94.6)	38 (100.0)	388 (95.1)		0.239^‡^
Married	20 (5.4)	0 (0.0)	20 (4.9)		
Living situation					
With family	114 (30.9)	12 (31.6)	126 (31.0)	0.22	0.896
In dormitory	14 (3.8)	2 (5.3)	16 (3.9)		
Alone	241 (65.3)	24 (63.2)	265 (65.1)		
Religion					
None	194 (52.4)	23 (60.5)	217 (53.2)		0.782^‡^
Christianity	106 (28.6)	11 (28.9)	117 (28.7)		
Catholicism	43 (11.6)	2 (5.3)	45 (11.0)		
Buddhism	24 (6.5)	2 (5.3)	26 (6.4)		
Other religions	3 (0.8)	0 (0.0)	3 (0.7)		
Subjective SES					
High	71 (19.2)	9 (23.7)	80 (19.6)	3.92	0.141
Middle	271 (73.2)	23 (60.5)	294 (72.1)		
Low	28 (7.6)	6 (15.8)	34 (8.3)		
Subjective pocket money					
Sufficient	104 (28.1)	12 (31.6)	116 (28.4)	0.97	0.615
Moderate	214 (57.8)	19 (50.0)	233 (57.1)		
Insufficient	52 (14.1)	7 (18.4)	59 (14.5)		

*SES* socioeconomic status

^†^Statistical significance test was done by Mann–Whitney *U* test

^‡^Statistical significance tests were performed using Fisher’s exact test

### Protective and risk factors associated with suicidal ideation: univariate analyses

High levels of depression and social anxiety were related to current suicidal ideation. Total scores on the BDI (except item 9) were significantly higher in the participants with suicidal ideation than in those with no suicidal ideation (*p* < 0.001). Similarly, total and three-domain SPIN scores were higher in students with suicidal ideation than in those without suicidal ideation (*p* < 0.001). Meanwhile, the protective factors exhibited opposite patterns. Total scores on the RSES were significantly lower in the students with suicidal ideation than in those without suicidal ideation (*p* < 0.001). Total ERS scores were also significantly lower in students with suicidal ideation than in those without suicidal ideation (*p* < 0.001). With regard to the level of social support, the Duke-UNC FSSQ total scores were significantly lower in the students with suicidal ideation than in those without suicidal ideation (*p* < 0.001). Further, it was found that both subscale scores of the Duke-UNC FSSQ were significantly lower in the students with suicidal ideation than those without suicidal ideation (Confidant Support: *p* < 0.001; Affective Support: *p* < 0.001). Table 2 summarizes the comparison of risk and protective factors according to suicidal ideation status.

**Table 2:** Group comparisons of risk and protective factors according to the presence of suicidal ideation

Variable	Suicidal ideation			*p*
	No Mean ± SD	Yes Mean ± SD	Total Mean ± SD	
Risk factors				
Depression				
BDI total score (except item 9)	6.0 ± 5.4	19.0 ± 8.8	7.4 ± 7.0	< 0.001
Social anxiety				
SPIN total score	15.0 ± 10.6	27.8 ± 12.0	16.2 ± 11.4	< 0.001
SPIN components				
Fear	6.2 ± 4.2	11.1 ± 4.6	6.7 ± 4.5	< 0.001
Physical symptoms	1.8 ± 2.4	4.5 ± 3.3	2.0 ± 2.6	< 0.001
Avoidance	7.0 ± 5.0	12.2 ± 5.8	7.5 ± 5.3	< 0.001
Protective factors				
Self-esteem				
RSES total score	32.0 ± 4.6	22.1 ± 6.4	31.2 ± 5.6	< 0.001
Ego-resiliency				
ERS total score	39.2 ± 5.6	33.3 ± 7.3	38.6 ± 6.0	< 0.001
Perceived social support				
Duke-UNC FSSQ total score	33.9 ± 5.8	25.1 ± 9.3	33.0 ± 6.7	< 0.001
Duke-UNC FSSQ components				
Confidant support	20.6 ± 4.0	15.1 ± 6.0	20.1 ± 4.6	< 0.001
Affective support	13.3 ± 2.2	10.0 ± 3.7	13.0 ± 2.6	< 0.001

Statistical significance tests were performed using the Mann–Whitney U test

*BDI* Beck Depression Inventory; *SPIN* Social Phobia Inventory; *RSES* Rosenberg Self-Esteem Scale; *ERS* Ego-Resiliency Scale; *Duke-UNC FSSQ* Duke-University of North Carolina Functional Social Support Questionnaire

### Impact of protective and risk factors on suicidal ideation: multivariate analyses

**Fig. 1:**
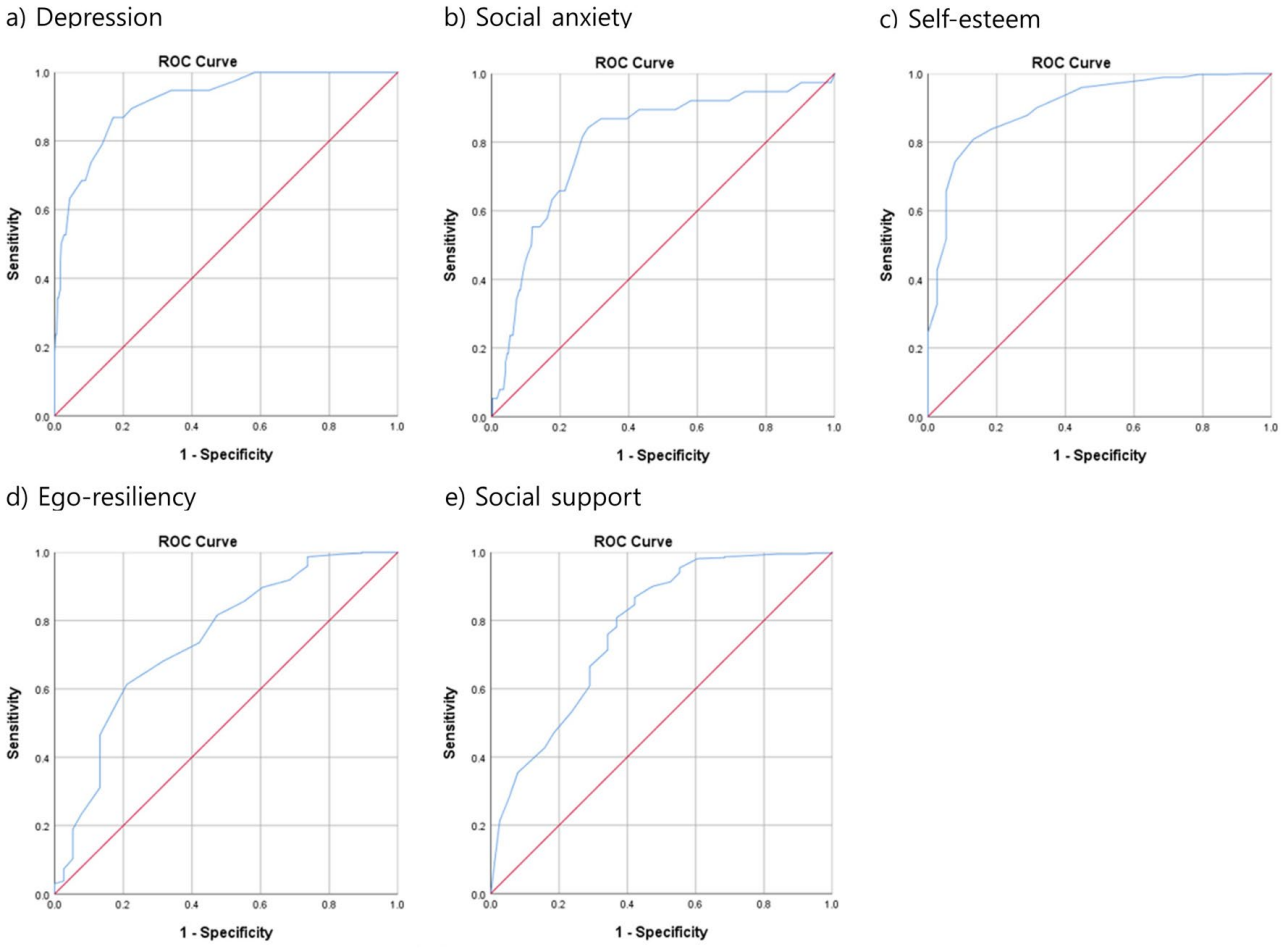
The ROC curves for depression (BDI except for item 9), social anxiety (SPIN), self-esteem (RSES), ego-resiliency (ERS), and social support (Duke-UNC FSSQ). **a** The ROC curve of BDI except for item 9 for suicidal ideation. AUC is 0.917 (95% CI [0.874–0.961]), *p* < 0.001. **b** The ROC curve of SPIN for suicidal ideation. AUC is 0.800 (95% CI [0.724–0.876]), *p* < 0.001. **c** The ROC curve of RSES for no suicidal ideation. AUC is 0.904 (95% CI [0.854–0.953]), *p* < 0.001. **d** The ROC curve of ERS for no suicidal ideation. AUC is 0.745 (95% CI [0.657–0.832]), *p* < 0.001. **e** The ROC curve of Duke-UNC FSSQ for no suicidal ideation. AUC is 0.777 (95% CI [0.692–0.863]). AUC area under the curve, BDI Beck Depression Inventory, SPIN Social Phobia Inventory, RSES Rosenberg Self-Esteem Scale, ERS Ego-Resiliency Scale, Duke-UNC FSSQ Duke-University of North Carolina Functional Social Support Questionnaire

The ROC curves for depression, social anxiety, self-esteem, ego-resiliency, and social support were significant, with AUCs of 0.917, 0.800, 0.904, 0.745, and 0.777, respectively (Fig. 1). The cut-off points for the BDI (not including item 9), SPIN, RSES, ERS, and FSSQ were 11.5 (11/12) with 86.8% sensitivity and 83.0% specificity, 18.5 (18/19) with 84.2% sensitivity and 71.9% specificity, 28.5 (28/29) with 80.8% sensitivity and 86.8% specificity, 37.5% (37/38) with 61.4% sensitivity and 78.9% specificity, and 26.5 (26/27) with 86.8% sensitivity and 57.9% specificity, respectively. Age (odds ratio [OR] = 0.954, 95% confidence interval [CI] [0.857–1.063], *p* = 0.396) had no significant effect on suicidal ideation in the first model and therefore was removed from the final model. Table 3 presents the final analysis model. The Hosmer–Lemeshow goodness-of-fit test confirmed the accuracy of the logistic model (*χ*^2^ = 2.911, *df* = 5, *p* = 0.714). In the final model, a higher level of depression (OR = 7.555, 95% CI [2.427–23.524], *p* < 0.001), social anxiety (OR = 3.085, 95% CI [1.065–8.934], *p* = 0.038), self-esteem (OR = 0.249; 95% CI [0.078–0.801], *p* = 0.020), and social support (OR = 0.341, 95% CI [0.142–0.818], *p* = 0.016) were independently associated with suicidal ideation. Meanwhile, a higher level of ego-resiliency (OR = 0.404, 95% CI [0.152–1.073], *p* = 0.069) showed a trend of association with suicidal ideation.

**Table 3:** Logistic regression model predicting suicidal ideation

Variable	*B*	*SE*	Wald	*p*	OR (95% CI)
Risk factors					
Depression, high	2.022	0.579	12.179	< 0.001	7.555 (2.427–23.524)
Social anxiety, high	1.127	0.543	4.312	0.038	3.085 (1.065–8.934)
Protective factors					
Self-esteem, high	− 1.389	0.596	5.435	0.020	0.249 (0.078–0.801)
Ego-resiliency, high	− 0.907	0.499	3.308	0.069	0.404 (0.152–1.073)
Social support, high	− 1.077	0.447	5.798	0.016	0.341 (0.142–0.818)

*χ*^2^ of model = 2.911, *df* = 5, Nagelkerke *R*^2^ = 0.525

### Relationship of ego-resiliency with risk factors on suicidal ideation

**Fig. 2:**
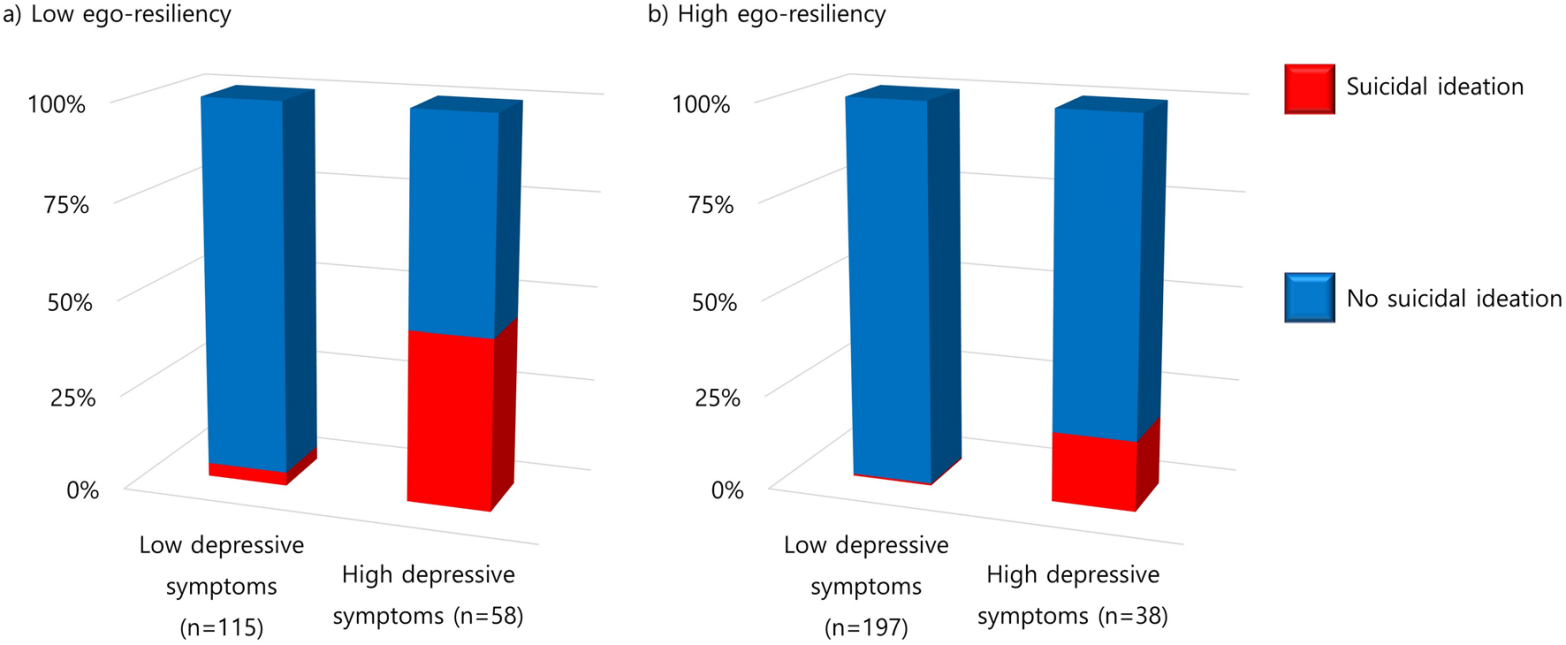
Interaction of ego-resiliency with depression on suicidal ideation. A significant interaction was found between ego-resiliency and depressive symptoms on suicidal ideation. In participants with high levels of depressive symptoms, the proportion of suicidal ideation was 44.8% (**a**) and 18.4% (**b**) when they had low and high ego-resiliency, respectively

**Fig. 3:**
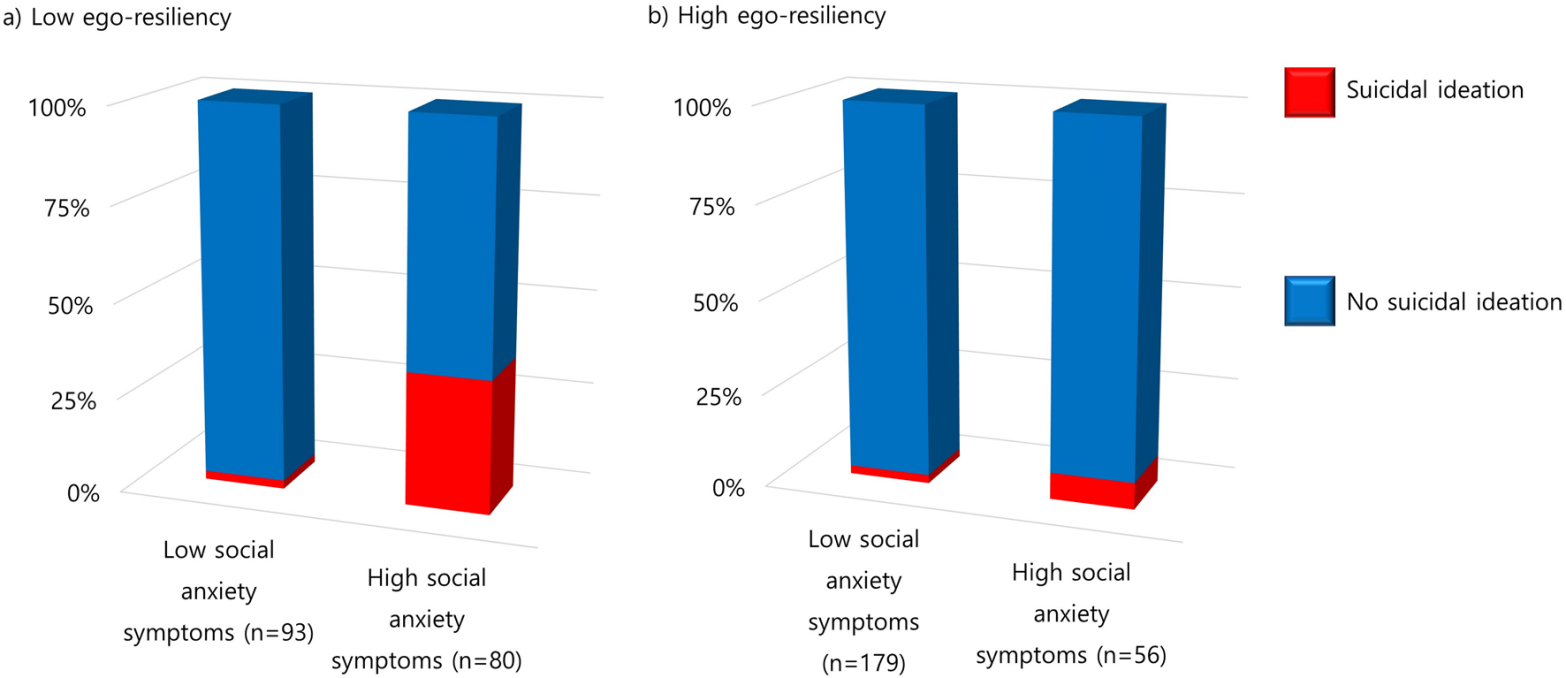
Interaction of ego-resiliency with social anxiety on suicidal ideation. A significant interaction was found between ego-resiliency and social anxiety symptoms on suicidal ideation. In participants with high levels of social anxiety symptoms, the proportion of suicidal ideation was 35.0% (**a**) and 7.1% (**b**) when they had low and high ego-resiliency, respectively

The potential moderating effects of ego-resiliency on the clinical risk factors of suicidal ideation were explored using CHAID. The interactions of ego-resiliency with both depression (*p* = 0.008) and social anxiety (*p* < 0.001) on suicidal ideation were significant. The pattern of the interaction between ego-resiliency and depression is shown in Fig. 2. Among participants with a lower level of ego-resiliency, 44.8% with a higher level of depression reported suicidal ideation. However, among students with a higher level of ego-resiliency, only 18.4% with a higher level of depression reported suicidal ideation. A similar pattern was observed in the interaction between ego-resiliency and social anxiety (Fig. 3). Furthermore, 35.0% of the participants with a higher level of social anxiety were found to have suicidal ideation when they had lower ego-resiliency. Meanwhile, only 7.1% of the participants with a higher level of social anxiety were found to have suicidal ideation when they had higher ego-resiliency.

## Discussion

Identifying the factors influencing suicidal ideation is important for suicide prevention. We examined the impact of potential protective and risk factors on suicidal ideation and its correlates in medical students. Our study revealed that medical students with higher levels of depression or social anxiety were more likely to have suicidal ideation, while those with higher levels of self-esteem or social support were less likely to have suicidal ideation. Although the independent effect of ego-resiliency on suicidal ideation did not reach statistical significance, it was found to moderate the risk of depression and social anxiety symptoms on suicidal ideation.

Our study demonstrated significant moderating effects of ego-resiliency between clinical risk factors and suicidal ideation. The medical students with suicidal ideation also showed a lower level of ego-resiliency than those without suicidal ideation. These results are in line with prior studies [33–35]. Besides ego-resiliency, factors related to psychological well-being, such as life satisfaction and happiness, have been found to play a protective role against depressive symptoms [36]. To date, few studies have investigated the moderating effect of ego-resiliency on suicide risk factors. The present study extends the previous findings to medical students, a population unstudied with regard to ego-resiliency. Our findings have several important implications for suicide prevention with medical students. First, the assessment of the level of ego-resiliency along with risk factors assists in differentiating those at higher risk of suicidality from those at lower risk. In addition, because resilience and positive emotions can be increased by psychological treatments [37], ego-resiliency and psychological well-being enhancement interventions can be applied to suicide prevention programs to strengthen the buffering effect. In our study, the protective effect of high ego-resiliency was significant in students with high depression and social anxiety symptoms. However, approaches that enhance ego-resiliency and positive emotions in those who suffer from severe psychiatric illness can be difficult owing to prominent negative cognitions and emotions. In this respect, well-timed and individualized approaches should be considered in the practical application of interventions addressing ego-resiliency.

Another internal component of protective factors, self-esteem, showed an inverse association with suicidal ideation in the final model; that is, medical students with higher levels of self-esteem had a reduced probability of experiencing suicidal ideation. In addition, a lower level of self-esteem was found to be significantly correlated with suicidal ideation. To the best of our knowledge, the association between self-esteem and suicidal ideation has not been investigated in medical students. Consistent with our findings, low self-esteem is reported as a significant predictor of suicidal ideation [38, 39]. Individuals with low self-esteem tend to dwell on unfavorable attributes rather than merits. This tendency of self-criticism may reach an extreme level, leading to suicidal ideation. Meanwhile, high self-esteem has been found to be a significant protective factor against depressed mood from adolescence to early adulthood [17]. Our results highlight the importance of self-esteem in the prevention of suicide, and special attention should be given to medical students with low self-esteem. Hence, specific and long-term plans to overcome low self-esteem need to be incorporated into suicide prevention programs in medical colleges.

Previous studies regarding the role of social support among medical students mainly focused on its impact on psychiatric symptoms but not on suicidality. In the present study, we found that social support was associated with a lower risk of suicidal ideation. In addition, medical students with suicidal ideation reported lower levels of social support than those without suicidal ideation. These findings are consistent with previous studies [19, 40–43]. Supportive relationships and environments could be valuable resources for medical students to cope with psychological distress during medical school. In line with these studies, our findings support the perspective that social support is negatively associated with suicidal ideation. Meanwhile, Min et al. [44] did not find this relationship, and Rospenda et al. [45] reported that higher levels of outside support were related to lower levels of academic performance among medical students. Thus, future studies are needed that examine the impact of specific types of social support on medical students.

The severity of depression was higher in medical students with suicidal ideation than in those without suicidal ideation. Further, a higher level of depression was the strongest predictor of suicidal ideation in the logistic regression analysis, indicating that underlying depression is an important risk factor for suicidal ideation. This finding is consistent with prior studies [40, 46]. In fact, recurrent suicidal ideation is a serious symptom of major depressive disorder (MDD). Of note, along with depression, burnout and fatigue were associated with a higher suicide risk in college students [46, 47]. Contrary to depression, there has been no research on the association between social anxiety and suicidal ideation among medical students. Previous epidemiological studies showed that social anxiety disorder (SAD) symptoms were associated with increased risk of suicidality [48, 49]. This higher risk of suicidal ideation may be due to increased isolation and hopelessness, since research suggests social anxiety is associated with social skills deficits as well as social withdrawal [50]. We found that medical students with higher levels of social anxiety had an increased risk of suicidal ideation. Our findings broaden the literature linking social anxiety and suicidal ideation in a clinical sample to medical students. Further, not only overall social anxiety but also all subdomains were found to be significant correlates of suicidal ideation. This suggests that each symptom aspect of social anxiety may affect suicidal ideation. Given that the risk of suicide attempts in SAD mainly results from comorbid disorders such as MDD [48], medical students with both disorders may be at greater risk of suicide. This point should be considered in suicide prevention programs in medical colleges.

The prevalence of suicidal ideation in our sample was 9.3%. This rate is higher than 7.2% reported among Brazilian medical students [40] and is similar to those of previous studies among medical students in the United States [46]. Meanwhile, this rate is lower than that found in samples from Norway, Sweden, and Pakistan, reporting rates of 14 to 35.6% [51–53]. These differences may result from the different lengths of time participants were asked about experiencing suicidal ideation. For example, longer timeframes (e.g., lifetime) would likely result in higher rates of suicidal ideation [54]. In addition, the different instruments may influence the rates reported. However, despite these methodological differences, a number of recent studies, except one from Pakistan, reported the last 1-year rates of suicidal ideation ranging from 7 to 11.7% [14]. In a meta-analysis of 24 studies, the pooled prevalence was 11.1% [7], which is comparable to our results evaluating current suicidal ideation. These findings suggest that a substantial number of medical students may experience suicidal ideation. Thus, active efforts to evaluate suicidal ideation and plans are required in medical colleges.

In our study, suicidal ideation was significantly more frequent in younger students, and the prevalence rate was highest in the first year. Furthermore, we found an association between earlier years of training and suicidal ideation, which is consistent with studies in Korea and Pakistan [53, 55]. In Korean medical colleges, most students receive premedical courses prior to their 4-year medical education. Compared with premedical education, a sudden increase in academic pressure and stress on failing in the first year may explain our results. On the contrary, third- and fourth-year students more frequently reported suicidal ideation than the first and second year students in the United States [46]. Medical students in the United States begin their medical education after obtaining a bachelor’s degree, which may contribute to the difference. Although a recent systematic review indicated the association between the year of study and suicidal ideation among medical students was inconsistent across studies [14], our findings imply that factors related to the year of medical education may be associated with suicidality. Future studies are needed to identify the specific risk factors associated with year of medical school education/training to better understand its relation to suicidal ideation.

Lower socioeconomic status has been reported to be significantly correlated with suicidal ideation among medical students [56, 57]. However, we did not find a relationship between socioeconomic status and suicidal ideation, which is consistent with findings by Torres et al. [40]. In fact, the present study evaluated the subjective level of socioeconomic status and pocket money rather than objective financial circumstances. Thus, the relationship between real financial difficulty in keeping up with the demands of medical school training and suicidality needs to be further investigated.

Despite the significant implications of the present study, some limitations and future directions should be discussed. First, because the design of this study was cross-sectional, it is not possible to determine causal inferences. A longitudinal study is warranted to confirm the causal relationship between protective/risk factors and suicidal ideation. In addition, future studies on intervention programs that strengthen ego-resiliency are required to verify the moderating effect of ego-resiliency in the relationship between clinical risk factors and suicidal ideation. Because the participants in our study were from one medical college, the sample may not be representative of all Korean medical students. Although the response rate was good, there is the potential for sampling bias because participant selection was performed based on the convenience method. We used item 9 on the BDI to assess current suicidal ideation; however, the one item cannot evaluate important aspects of suicidal ideation such as preparedness and lethality of the method. Further investigation into a detailed plan or method would be helpful to elaborate the factors associated with later suicidal behavior. Unfortunately, we did not assess major issues related to suicidality among medical students, including substance abuse, dissatisfaction with academic performance, and parental neglect. Additional studies on these issues may broaden our knowledge of the correlates of suicidal ideation among medical students. Prior to the construction of the regression model, the continuous variables of protective and risk factors were transformed into dichotomous variables, which might be useful for the clinical application of our findings. However, this approach necessarily involves a loss of original data and the problem of arbitrary cut-off scores. Lastly, the results should be interpreted with caution because of the limitation of reliance on self-report measures.

## Conclusion

In conclusion, our findings indicated that higher levels of self-esteem and social support were associated with a lower risk of suicidal ideation, whereas higher levels of depression and social anxiety were related to a higher risk of suicidal ideation among medical students. Ego-resiliency was found to moderate the risk of depression and social anxiety on suicidal ideation, suggesting a meaningful buffering role against suicidality. In addition, this study identified the current status of and factors related to suicidal ideation among medical students in Korea. On the basis of these findings, particular attention should be given to medical students with both depression and social anxiety. Further, our study suggests that integrative mental health programs focusing on promoting ego-resiliency, self-esteem, and social support may contribute to suicide prevention in medical students.

## Data Availability

Not applicable.

## Abbreviations

OECD: Organisation for Economic Cooperation and Development
BDI: Beck Depression Inventory
SPIN: Social Phobia Inventory
RSES: Rosenberg Self-Esteem Scale
ERS: Ego-Resiliency Scale
Duke-UNC FSSQ: Duke-University of North Carolina Functional Social Support Questionnaire
ROC: Receiver operating characteristic
AUC: Area under the curve
CHAID: Chi-square automatic interaction detection
OR: Odds ratio
CI: Confidence interval
MDD: Major depressive disorder
SAD: Social anxiety disorder

## References

[1] World Health Organization (2014). Preventing suicide: a global imperative.

[2] Organization for Economic Cooperation and Development (OECD). OECD data: suicidal rate https://data.oecd.org/healthstat/suicide-rates.htm Accessed 31 Oct 2020.

[3] Bostwick JM, Pabbati C, Geske JR, McKean AJ (2016). Suicide attempt as a risk factor for completed suicide: even more lethal than we knew. Am J Psychiatry.

[4] Beck AT (1986). Hopelessness as a predictor of eventual suicide. Ann N Y Acad Sci.

[5] Park EH, Hong N, Jon DI, Hong HJ, Jung MH (2017). Past suicidal ideation as an independent risk factor for suicide behaviours in patients with depression. Int J Psychiatry Clin Pract.

[6] Garlow SJ, Rosenberg J, Moore JD, Haas AP, Koestner B, Hendin H (2008). Depression, desperation, and suicidal ideation in college students: results from the American foundation for suicide prevention college screening project at emory university. Depress Anxiety.

[7] Rotenstein LS, Ramos MA, Torre M, Segal JB, Peluso MJ, Guille C (2016). Prevalence of depression, depressive symptoms, and suicidal ideation among medical students: a systematic review and meta-analysis. JAMA.

[8] Hays LR, Cheever T, Patel P (1996). Medical student suicide, 1989–1994. Am J Psychiatry.

[9] Dyrbye LN, Thomas MR, Shanafelt TD (2006). Systematic review of depression, anxiety, and other indicators of psychological distress among U.S. and Canadian medical students. Acad Med.

[10] Tjia J, Givens JL, Shea JA (2005). Factors associated with undertreatment of medical student depression. J Am Coll Health.

[11] Ratnani IJ, Vala AU, Panchal BN, Tiwari DS, Karambelkar SS, Sojitra MG (2017). Association of social anxiety disorder with depression and quality of life among medical undergraduate students. J Family Med Prim Care.

[12] Al-Hazmi BH, Sabur SS, Al-Hazmi RH (2020). Social anxiety disorder in medical students at Taibah University, Saudi Arabia. J Family Med Prim Care.

[13] Davidson CL, Wingate LR, Grant DM, Judah MR, Mills AC (2011). Interpersonal suicide risk and ideation: the influence of depression and social anxiety. J Soc Clin Psychol.

[14] Coentre R, Góis C (2018). Suicidal ideation in medical students: recent insights. Adv Med Educ Pract.

[15] Oquendo MA, Currier D, Mann JJ (2006). Prospective studies of suicidal behavior in major depressive and bipolar disorders: what is the evidence for predictive risk factors?. Acta Psychiatr Scand.

[16] Block J, Kremen AM (1996). IQ and ego-resiliency: conceptual and empirical connections and separateness. J Pers Soc Psychol.

[17] Costello DM, Swendsen J, Rose JS, Dierker LC (2008). Risk and protective factors associated with trajectories of depressed mood from adolescence to early adulthood. J Consult Clin Psychol.

[18] Taylor ZE, Jones BL (2020). Cultural contributors to ego-resiliency and associations with depressive problems in Midwestern Latino youth. J Res Adolesc.

[19] Pietrzak RH, Goldstein MB, Malley JC, Rivers AJ, Johnson DC, Southwick SM (2010). Risk and protective factors associated with suicidal ideation in veterans of operations enduring freedom and Iraqi freedom. J Affect Disord.

[20] Seo EH, Kim SG, Lee SK, Park SC, Yoon HJ (2021). Internet addiction and its associations with clinical and psychosocial factors in medical students. Psychiatry Investig.

[21] Beck AT, Ward CH, Mendelson M, Mock J, Erbaugh J (1961). An inventory for measuring depression. Arch Gen Psychiatry.

[22] Shin MS, Kim ZS, Park KB (1993). The cut-off score for the Korean version of beck depression inventory. Korean J Clin Psychol.

[23] Connor KM, Davidson JR, Churchill LE, Sherwood A, Foa E, Weisler RH (2000). Psychometric properties of the Social phobia inventory (SPIN). New self-rating scale Br J Psychiatry.

[24] Cho Y, Choi Y, Kim S, Hong S (2018). Factor structure and other psychometric properties of the social phobia inventory in Korean samples. Meas Eval Couns Dev.

[25] Rosenberg M (1965). Society and the adolescent self-image.

[26] Bae HN, Choi SW, Yu JC, Lee JS, Choi KS (2014). Reliability and validity of the Korean version of the Rosenberg self-esteem scale (K-RSES) in adult. Mood Emot.

[27] Yoo SK, Shim HW (2002). Psychological protective factors in resilient adolescents in Korea. Korean J Educ Psychol.

[28] Broadhead WE, Gehlbach SH, de Gruy FV, Kaplan BH (1988). The Duke-UNC functional social support questionnaire. Measurement of social support in family medicine patients. Med Care.

[29] Broadhead WE, Kaplan BH (1991). Social support and the cancer patient. Implications for future research and clinical care. Cancer.

[30] Suh SY, Im YS, Lee SH, Park MS, Yoo T (1997). A study for the development of Korean version of the Duke-UNC Functional Social Support Questionnaire. J Korean Acad Fam Med.

[31] Zou KH, Hall WJ, Shapiro DE (1997). Smooth non-parametric receiver operating characteristic (ROC) curves for continuous diagnostic tests. Stat Med.

[32] Kass GV (1980). An exploratory technique for investigating large quantities of categorical data. J Appl Stat.

[33] Park S, Bak E, Lee S, Jang A, Cho S (2016). Effects of social support, ego-resilience, and subjective wellbeing on suicidal ideation in nursing students. J Korean Acad Fundam Nurs.

[34] Cha KS, Lee HS (2018). The effects of ego-resilience, social support, and depression on suicidal ideation among the elderly in South Korea. J Women Aging.

[35] Nrugham L, Holen A, Sund AM (2010). Associations between attempted suicide, violent life events, depressive symptoms, and resilience in adolescents and young adults. J Nerv Ment Dis.

[36] Seo EH, Kim SG, Kim SH, Kim JH, Park JH, Yoon HJ (2018). Life satisfaction and happiness associated with depressive symptoms among university students: a cross-sectional study in Korea. Ann Gen Psychiatry.

[37] Fava GA, Tomba E (2009). Increasing psychological well-being and resilience by psychotherapeutic methods. J Pers.

[38] Wilburn VR, Smith DE (2005). Stress, self-esteem, and suicidal ideation in late adolescents. Adolescence.

[39] Jang JM, Park Jl OhKY, Lee KH, Kim MS, Yoon MS (2014). Predictors of suicidal ideation in a community sample: roles of anger, self-esteem, and depression. Psychiatry Res.

[40] Torres AR, Campos LM, Lima MCP, Ramos-Cerqueira ATA (2018). Suicidal ideation among medical students: prevalence and predictors. J Nerv Ment Dis.

[41] Moak ZB, Agrawal A (2010). The association between perceived interpersonal social support and physical and mental health: results from the national epidemiological survey on alcohol and related conditions. J Public Health.

[42] Jeong Y, Kim JY, Ryu JS, Lee KE, Ha EH, Park H (2010). The associations between social support, health-related behaviors, socioeconomic status and depression in medical students. Epidemiol Health.

[43] Bomyea J, Lang AJ, Craske MG, Chavira D, Sherbourne CD, Rose RD (2013). Suicidal ideation and risk factors in primary care patients with anxiety disorders. Psychiatry Res.

[44] Min JA, Lee CU, Chae JH (2015). Resilience moderates the risk of depression and anxiety symptoms on suicidal ideation in patients with depression and/or anxiety disorders. Compr Psychiatry.

[45] Rospenda KM, Halpert J, Richman JA (1994). Effects of social support on medical students’ performances. Acad Med.

[46] Dyrbye LN, Thomas MR, Massie FS, Power DV, Eacker A, Harper W (2008). Burnout and suicidal ideation among U.S. medical students. Ann Intern Med.

[47] Nyer M, Mischoulon D, Alpert JE, Holt DJ, Brill CD, Yeung A (2015). College students with depressive symptoms with and without fatigue: differences in functioning, suicidality, anxiety, and depressive severity. Ann Clin Psychiatry.

[48] Schneier FR, Johnson J, Hornig CD, Liebowitz MR, Weissman MM (1992). Social phobia. Comorbidity and morbidity in an epidemiologic sample. Arch Gen Psychiatry.

[49] Gallagher M, Prinstein MJ, Simon V, Spirito A (2014). Social anxiety symptoms and suicidal ideation in a clinical sample of early adolescents: examining loneliness and social support as longitudinal mediators. J Abnorm Child Psychol.

[50] Beidel DC, Rao PA, Scharfstein L, Wong N, Alfano CA (2010). Social skills and social phobia: an investigation of DSM-IV subtypes. Behav Res Ther.

[51] Tyssen R, Vaglum P, Grønvold NT, Ekeberg O (2001). Suicidal ideation among medical students and young physicians: a nationwide and prospective study of prevalence and predictors. J Affect Disord.

[52] Dahlin M, Joneborg N, Runeson B (2005). Stress and depression among medical students: a cross-sectional study. Med Educ.

[53] Osama M, Islam MY, Hussain SA, Masroor SM, Burney MU, Masood MA (2014). Suicidal ideation among medical students of Pakistan: a cross-sectional study. J Forensic Leg Med.

[54] Amiri L, Voracek M, Yousef S, Galadari A, Yammahi S, Sadeghi MR (2013). Suicidal behavior and attitudes among medical students in the United Arab Emirates. Crisis.

[55] Roh MS, Jeon HJ, Kim H, Han SK, Hahm BJ (2010). The prevalence and impact of depression among medical students: a nationwide cross-sectional study in South Korea. Acad Med.

[56] Fan AP, Kosik RO, Mandell GA, Tran DT, Cheng HM, Chen CH (2012). Suicidal ideation in medical students: who is at risk?. Ann Acad Med Singap.

[57] Coentre R, Faravelli C, Figueira ML (2016). Assessment of depression and suicidal behaviour among medical students in Portugal. Int J Med Educ.

